# Non-Convulsive Status Epilepticus as A Complication of Electroconvulsive Therapy: A Case Report

**DOI:** 10.1192/j.eurpsy.2023.570

**Published:** 2023-07-19

**Authors:** T. Saltoglu, B. Senol, G. Koc

**Affiliations:** ^1^Neurology; ^2^Psychiatry, Ankara City Hospital, Ankara, Türkiye

## Abstract

**Introduction:**

Status Epilepticus is defined as a condition that can have long-term outcomes involving neuronal death and injury due to the failure of the mechanisms responsible for seizure termination or from the initiation of mechanisms that lead to abnormally prolonged seizures. Electroconvulsive therapy (ECT) is a highly effective treatment option for psychiatric disorders. Although it rarely occurs in the treatment, non-convulsive status epilepticus can be seen as a complication after ECT. Due to its rarity, this complication is not yet well understood, is challenging to diagnose, and information about treatment options is limited.

**Objectives:**

By sharing this case report, we aim to emphasize the importance of being careful in terms of the risk of status epilepticus in patients receiving electroconvulsive therapy.

**Methods:**

Here in we present a 29-year-old patient with no previous neurological disease and who had a history of schizophrenia. Electroconvulsive therapy was planned because the patient was resistant to antipsychotic treatment. EEG was planned for the patient who had urinary incontinence during the ninth session of ECT. Generalized slow wave activity and intermittent rhythmic delta activity were observed in the EEG, therefore it was found suspicious for NCSE, and the patient was planned to perform an EEG again by administering diazepam to confirm the diagnosis. After diazepam, the patient whose EEG tracing was clearly improved was admitted to the neurology intensive care unit. He was followed up for 48 hours with continuous 4 mg/hour/day midazolam and continuous bedside EEG in the neurology intensive care unit. Concomitant lamotrigine was started at 100 mg/day. Significant improvement in EEG, sinusoidal alpha, and beta waves with the eye open was observed at the 48th hour, and the patient was transferred back to the psychiatry service. Lamotrigine treatment was increased up to 200 mg/day and clozapine treatment was adjusted to 350 mg/day in the psychiatry service. In the patient whose EEG was requested again before discharge.

**Results:**

The diagnosis of NCSE post-ECT can be laborious; the symptoms may not be characteristic and clear, and usually not distinguish from symptoms of confusion, delirium, or psychiatric illness, hence the follow-up psychiatrist should be careful. In suspicious cases, EEG should be taken, especially in patients at risk for seizures. These risky conditions include previous seizure history, and lithium or clozapine use.

**Conclusions:**

The diagnosis of NCSE after ECT is a demanding condition. Particular attention should be paid to factors that will lower the seizure threshold. In cases with ECT treatment with clozapine, intermittent clozapine blood levels can be quantified and medication interactions and smoking can be considered. When the cases are examined, the common aspect of most of them is that the treatments have good results.

**Disclosure of Interest:**

None Declared

